# Cerebral small vessel disease, systemic vascular characteristics and potential therapeutic targets

**DOI:** 10.18632/aging.203557

**Published:** 2021-09-22

**Authors:** Salim Elyas, Damilola Adingupu, Kunihiko Aizawa, Francesco Casanova, Kim Gooding, Jonathan Fulford, Dave Mawson, Phillip E. Gates, Angela C. Shore, David Strain

**Affiliations:** 1 Institute of Biomedical and Clinical Science and NIHR Exeter Clinical Research Facility, University of Exeter Medical School, Exeter EX2 5AX, UK; 2 Academic Department of Healthcare for Older People, Royal Devon and Exeter NHS Foundation Trust, Exeter EX2 5DW, UK

**Keywords:** cerebral small vessel disease, white matter hyperintensities, leucoaraiosis, vascular markers, dementia

## Abstract

Introduction: Cerebral small vessel disease (SVD) is prevalent in the elderly population and is associated with increased risk of dementia, stroke and disability. Currently there are no clear targets or strategies for the treatment of cerebral SVD. We set out to identify modifiable vascular treatment targets.

Patients and Methods: 112 participants with and without a history of CVD underwent macrovascular, microvascular and endothelial function tests and an MRI head scan.

Results: Increased carotid intima media thickness and carotid-femoral pulse wave velocity were associated with cerebral WMH (β=1·1 p=0·001 and β=1·66, p<0·0001 respectively). Adjusted cerebral resistance index (p=0·03) and brachial flow mediated dilation time to peak (p=0·001) were associated with the severity of cerebral WMH independent of age and sex. Post occlusive reactive hyperaemia time as a measure of microvascular reactivity was associated with WMH after adjustment for age and sex (p=0·03). Ankle Brachial Pressure Index and urinary albumin excretion rate predicted the severity of cerebral WMH (p=0·02 and 0·01 respectively). Age and hypertension were the most important risk factors for WMH severity (p< 0·0001).

Discussion: In addition to hypertension, microalbuminuria, arterial stiffness, vascular reactivity and cerebrovascular resistance could be potential treatment targets to halt the development or progression of cerebral SVD.

## INTRODUCTION

Cerebral small vessel disease (SVD) is a leading cause of cognitive decline and functional loss in the elderly [1]. Cerebral SVD is recognised by the resultant parenchymal lesions rather than the underlying small vessel alterations themselves, and typically manifests as lacunar lesions, diffuse white matter lesions (leucoaraiosis) and/or microbleeds. Accordingly, cerebral white matter hyperintensities (WMH) on MRI scan are recognised surrogates of cerebral SVD [1]. Although the aetiopathogenic mechanisms of cerebral SVD are unclear, there is a clear distinction from cerebral large vessel disease. Indeed, with the exception of hypertension, conventional cardiovascular risk factors such as diabetes and hyperlipidaemia have inconsistent correlation with cerebral SVD [2–5]. Further, after accounting for age and the traditional vascular risk factors, much of the variance in WMH volume remains unexplained [6].

Cerebral WMH predict incident stroke, dementia, heart failure, disability and mortality [1, 7]. Despite the lack of correlation with conventional cardiovascular risk factors and with no supporting evidence base, current treatment strategies for managing cerebral WMH are extrapolated from the general management guidelines for the treatment of atherosclerotic disease. As a result, treatment strategies focusing on the use of antiplatelet, statins, aggressive blood pressure (BP) lowering and (in people with diabetes) aggressive glycaemic control may not be effective in the treatment of cerebral SVD [8, 9]. Indeed, the use of dual antiplatelet therapy is associated with a significantly increased risk of bleeding, whilst excessive BP lowering to targets of <130 mmHg is associated with cognitive decline in patients with cerebral SVD [10, 11]. The absence of specific treatments for cerebral SVD precipitates the need for clear targets or strategies for the treatment of cerebral SVD.

We propose the search for such targets should focus on the underlying pathology of cerebral SVD, focusing specifically on the regulation of cerebral microcirculation. However, in order to identify potential treatment strategies, knowledge of the systemic correlates of cerebral SVD is required. As WMH are present in patients with and without history of previous CVD, we aimed to explore this in a general population sample of older adults enriched with patients with proven cerebral SVD.

## RESULTS

Of 169 potential participants screened, 57 were unable to undergo MRI (predominantly due to permanent pacemaker placement, surgical metal work or claustrophobia). The study therefore yielded 112 participants with a full data set. The population characteristics are shown in Table 1. There were more males in the study (77 males vs. 35 female), however, there was no difference in WMH volume between the sexes (p=0.7).

**Table 1:** Population characteristics of study participants.

**Population characteristics**	
**Gender**	Males 77 (69%)
	Females 35 (31%)
Age (years)	68 ± 9
Systolic Blood pressure (mmHg)	130 ± 13
Diastolic blood pressure (mmHg)	74 ± 8
Diabetes	7 (6%)
Treated hypertension	56 (50%)
Ischaemic heart disease	13 (12%)
Previous transient ischaemic attack	44 (39%)
Previous stroke	31 (28%)
Smoking	60 (54%)
Alcohol > 15 units/week	25 (22%)
Statins	74 (66%)
Calcium channel blockers	18 (16%)
ACE inhibitors	28 (25%)
Beta blockers	11 (10%)
Diuretics	22 (20%)
Nitrates	1 (0.9%)
Body Mass Index (kg/m^2^)	27 ± 4
White matter hyper-intensities load (cm^3^) (IQ Range)	3·65 (2·0 – 6·0)

### Association between vascular risk factors and WMH volume

In univariate analysis, as anticipated, WMH volume was significantly higher in participants with history of hypertension compared to non-hypertensive individuals (4·5 cm^3^ (CI: 3·7 – 5·3) cm^3^ vs. 2·8 (CI: 2·3 – 3·4) cm^3^, p<0·0001). Further, aortic systolic BP and MAP (but not diastolic BP), aortic pulse pressure, ABPI were independently associated with WMH volume after adjustment for age and sex. Unsurprisingly, those recruited from stroke clinic had a higher WMH volume than the general population sample (4·4 (CI: 3·6 – 5·3) cm^3^ vs. 2·6 (CI 2·1 – 3·2) cm^3^, p=0·001).

Cerebral WMH volume was associated with urinary albumin excretion rate, independent of age and sex. This was independent of diabetes, which only demonstrated a non-significant trend towards an association (5·3 (CI: 3·1 – 9·1) cm^3^ vs. 3·5 (CI: 3·0 – 4·0) cm^3^ for those with and without diabetes respectively; p=0·09). There was no significant difference in the volume of WMH between smokers and non-smokers (3·4 (CI: 2·9 – 4·1) cm^3^ vs. 3·7 (CI: 3·0 – 4·2) cm^3^ respectively; p=0·8).

There was a paradoxical association between cholesterol and WMH, such that lower cholesterol was associated with higher WMH volume, but this is likely confounded by statin use in the population recruited from stroke clinic (Table 2). Of these variables, ABPI and urinary AER showed a trend towards predicting WMH after adjustment for aortic MAP, diabetes, lipids, smoking and alcohol volume (ß= -0·78, p=0·07 and ß= 0·17, p=0·08 respectively).

**Table 2:** Linear regression analysis of vascular risk factors as predictors of WMH volume adjusted for age and sex.

**Parameter**	**ß**	**95% CI**	**R^2^ (%)**	**P value**	**P value adjusted for age and sex**
Age	0·04	0·02 – 0·06	22	<0·0001	
Systolic BP (mmHg)*	0·02	0·01 – 0·04	15	<0·0001	0·001
Diastolic BP (Age ≥ 70) (mmHg)*	0·03	- 0·01 – 0·06	5	0·14	0·09
Diastolic BP (Age < 70) (mmHg)*	0·03	0·01 – 0·04	10	0·007	0·008
Mean Arterial Pressure (mmHg)*	0·025	0·01 – 0·04	8	0·003	0·001
Pulse pressure (mmHg)*	0·033	0·02 – 0·05	17	<0·0001	0·004
Ankle Brachial Pressure Index (ABPI)	- 1·17	(- 2·1) – (-0·23)	6	0·02	0·04
Weight (kg)	0·003	0·008 – 0·015	0·3	0·6	0·1
Body Mass Index (kg/m^2^)	0·016	- 0·02 – 0·05	0·7	0·4	
Waist Hip Ratio (cm)	1·9	0·19 – 3·6	4	0·03	0·16
Fasting glucose (mmol/L)	0·05	- 0·1 – 0·2	0·5	0·5	
HbA1c (mmol/L)	0·005	- 0·03 – 0·04	0·08	0·8	
Total Cholesterol (mmol/L)	-0·14	-0·028 – 0·005	3	0·06	0·02
Plasma viscosity (mPa.s)	-0·12	- 2·4 – 2·2	0·01	0·9	
Alcohol (units per week)	-0·007	- 0·03 – 0·01	0·4	0·5	
Creatinine (μmol/L)	0·5	- 0·18 – 1·2	0·25	0·01	0·14
Albumin Excretion Rate (μg/min)	0·27	0·066 – 0·47	6·5	0·01	0·05

ß, Beta regression coefficient; R^2^, variance explained by model, ABPI, Ankle brachial pressure index; *, central aortic pressure.

### Association between vascular measurements and WMH volume

As anticipated, a lower cerebral EDV was associated with increasing WMH volume (β= -0·9, p=0·02). However, this association was accounted for by age and sex (p=0·2). Cerebral PI and RI were associated with WMH (β=2·4 (CI: 1·3 – 3·6), p<0·0001 and β=6·0 (CI: 3 – 9). P<0·0001 respectively) but this association was also attenuated by age and sex (p= 0·07 and 0·14 respectively). ACRI, which accounts for differences in aortic BP, however, was positively associated with WMH volume independent of age and sex (β=0·9 (CI: 0·08 – 1·6), p =0·03, adjusted β=0·72 (CI: -0·0004 – 1·4), p=0·05).

Carotid-femoral PWV (β=1·66 (CI: 0·97 – 2·3), p< 0·0001) and carotid IMT (β=1·1 (CI: 0·42 – 1·7), p=0·001) were associated with WMH volume, although the latter of these was attributable to age and sex (p after adjustment=0·08).

Brachial FMD and time to achieve maximal dilation, were both associated with WMH volume such that attenuated dilation and an increased time to achieve maximum dilation were associated with higher WMH volume. (β= -0·19 (CI: -0·37– (-0·003)), p=0·05 and 0·9 (CI: 0·4 – 1·4), p=0·001 respectively). However, only time to achieve maximum dilation bore adjustment for age, sex (Adjusted β=0·65 (CI: 0·2 – 1·1, p=0·004).

Similarly, and after adjustment for age and sex, a longer time to achieve microvascular peak PORH was associated with a higher WMH volume (Beta regression coefficient after adjustment = 0·3 (CI: 0·03 – 0·6), p=0·03). This was also independent of central MAP β=0·35 (CI: 0·08 – 0·63; p=0·012). No other measure of microvascular function or endothelial independent response predicted WMH volume (Table 3).

**Table 3:** Regression analysis of vascular measurements as predictors of WMH volume (unadjusted and adjusted to age and sex).

**Parameter**	**ß**	**95% CI**	**R^2^**	**P value**	**ß adjusted for age and sex**	**P value adjusted for age, sex**
Carotid Femoral PWV (m/s)	1·66	0·97 – 2·3	0·18	<0·0001	1·0	0·01
Intima Media Thickness (mm)	1·1	0·42 – 1·7	0·1	0·001	0·6	0·08
Cerebral PSV (m/s)	0·02	- 0·08 – 0·88	0	1·0		
Cerebral EDV (m/s)	-0·9	- 1·7 – (-0·12)	0·05	0·02	-0·5	0·2
Cerebral MFV (m/s)	-0·5	- 1·4 – 3·5	0·014	0·25		
Cerebral PI	2·4	1·3 – 3·6	0·16	<0·0001	1·3	0·07
Cerebral RI	6·0	3 – 9	0·14	<0·0001	2·7	0·14
Adjusted Cerebral Resistance Index (ACRI)	0·9	0·08 – 1·6	0·05	0·03	0·7	0·05
Flow Mediated Dilation (%)	-0·19	-0·37– (-0·003)	0·04	0·05	-0·07	0·4
FMD Time to Peak (s)	0·9	0·4 – 1·4	0·14	0·001	0·65	0·004
Ach Peak Response (flux units)	0·3	- 0·4 – 1·0	0·01	0·4		
Ach AUC	0·15	- 0·3 – 0·6	0·005	0·5		
PORH peak response	-0·09	-0·4 – 0·23	0·003	0·6		1.0
PORH Time to Peak (s)	0·18	-0·15 – 0·5	0·01	0·3	0·3	0·03
PORH hyperaemia duration (s)	-0·02	-0·3 – 0·26	0·0002	0·9		0·4

ß, Beta regression coefficient; R^2^, variance explained by model; PWV, pulse wave velocity; PSV, peak systolic velocity; EDV, end diastolic velocity; MFV, mean flow velocity; PI, Pulsatility index; RI, resistive index; FMD, flow mediated dilation; Ach, Acetylcholine; AUC, area under curve; PORH, post occlusive reactive hyperaemia.

## DISCUSSION

In this study we explored the microvascular and macrovascular function of subjects with a range of cerebral SVD which is arguably different from the classic atherosclerotic picture seen in patient with large vessel disease and indeed could lend further support to the different physiological and epidemiological profiles of SVD and large vessel disease. This is characterised by a clear association between cerebral WMH and measures of microcirculatory autoregulation, namely cerebral vascular resistance. Further, there was an association with urinary AER, and both cerebral and peripheral microvascular reactivity. There was, however, no association between classic measures of endothelial function and cerebral WMH. This is contrary to findings in large vessel disease, suggesting a potential different mechanistic pathway of large vessel and small vessel disease [12]. It may also help to explain the lack of benefit seen in those with cerebral SVD when treated with therapies proven in large vessel disease and extrapolated onto this different disease process. As previously demonstrated, cerebral WMH was also tightly associated with BP and age [2]. This is in keeping with the reported benefit of good BP control in slowing the progression of cerebral WMH.

The association of WMH with urinary AER is consistent with other studies [13, 14]. Microalbuminuria has also been shown to predict high risk transient ischaemic attacks [15]. For this reason therapies such as ACE inhibitors, angiotensin receptor antagonists or direct renin inhibitors, which are known to reduce microalbuminuria should be considered in order to reduce stroke risk [16]. In the general population, there is no additional benefit from a treatment regime based on ACE-inhibition versus calcium channel blocker or thiazide type diuretic [17]. This treatment strategy, however has not been formally tested for the management of cerebral small vessel disease. and requires further exploration [18].

The association of carotid IMT, accepted as a surrogate for atherosclerosis, with cerebral WMH was attenuated after adjustment for age and sex, contrary to what has been demonstrated elsewhere [8]. This could well be the effect of the relatively small study sample size, however, this is in keeping with the lack of benefit from statin therapy, the cornerstone of anti-atherosclerotic disease progression, seen in large studies of people with cerebral WMH [19]. Conversely, ABPI includes several contributions from macrovascular and microvascular function which probably explains its association with cerebral WMH demonstrated here.

This is the first study that used the recognised surrogate markers of cutaneous microvascular function in patients with cerebral WMH. The association between systemic microvascular dysfunction, [impaired skin microvascular reactivity (PORH)], and cerebral WMH, provides additional evidence to support the suggestion that cerebral SVD is part of a systemic microvascular process [20, 21]. Further, this microcirculatory dysfunction may be part of a process eventually leading to end organ damage [20, 22, 23]. It is unlikely, however, that either process is completely independent, rather that end-organ damage is a result of a synergistic detrimental effect of microvascular and macrovascular disease precipitating both structural and functional changes. A greater knowledge of the longitudinal nature of these associations, and the interactions between the large and small vessels, will help determine appropriate treatment strategies focused on reducing progression of endothelial and microvascular dysfunction [24]. Isolating these novel targets may enable the slowing of pathogenic steps that lead to cerebral SVD and the subsequent adverse consequences on quality and quantity of life.

### Study strengths and limitations

Our study strengths include the comprehensive assessment of the macrovascular, microvascular and endothelial function, never before performed in a single population. Further, we used automated volumetric assessment of the WMH volume which has accurate data and does not have a ceiling effect. The study limitations include a relatively small study population that could contribute to underpowered results, particularly in those results where the p value was borderline. A lack of humoral markers of endothelial and vascular function that would have added another dimension to this study is another limitation of this study. Further, the study participants were mainly recruited from two specific sources (TIA clinic/stroke unit and the Exeter 10,000 cohort) which could have inherently led to selection bias. Cerebral SVD is an umbrella term for a number of conditions and imaging findings (WMH, lacunar strokes, micro-bleeds and perivascular spaces). In this study we mainly characterised WMH using the Freesurfer software which makes the results not generalizable to other forms of cerebral SVD. Another limitation of the study is the lack of dynamic cerebrovascular function indices (such as the breath holding test), this could have potentially added further information and helped better understand the functional reactivity of the cerebral circulation in patients with cerebral SVD, however the experience at our centre showed that these techniques are not well tolerated particularly by the older population.

## CONCLUSIONS

Patients with cerebral WMH have a poor vascular function profile. This is the first study to undertake a comprehensive evaluation of the function of the vascular tree and its association with cerebral WMH. We identified micro and macro vascular markers that are associated with cerebral WMH. Longitudinal work is required to determine whether modulation of these parameters can slow or even halt progression of cerebral SVD.

## MATERIALS AND METHODS

### Subjects

The study protocol was developed in line with the Helsinki declaration revised 1983. The protocol was approved by the local ethics committee (Southwest local ethics committee, UK). Patients with history of TIA and Stroke were recruited from the TIA clinic and the stroke unit (The Royal Devon and Exeter Hospital). A general population sample (age and gender matched) was recruited from a research register (the Exeter 10,000) of individuals from the Exeter community who volunteered to take partpart in research. This was to generate a study population with a range of cerebral small vessel disease with and without history of TIA/Stroke.

All participants gave written, informed consent and completed a questionnaire on demographics, medical history, medications and lifestyle behaviours. Participants who had a known contraindication for MRI were excluded from the study. Anthropometry including height, weight, ankle brachial pressure index (ABPI) and waist hip ratio was measured according to a standard protocol. Blood and urine samples were analysed by the on-site laboratory, which is a member of the appropriate UK National Quality Assessment Scheme.

At a separate visit macrovascular, microvascular and endothelial function were assessed after an overnight fast, from 10 pm the night before. All participants were requested to refrain from consuming coffee, tea, other caffeinated drinks, alcohol, medication and strenuous exercise to minimise the variance on vascular studies. All studies commenced at 9am after a standardised breakfast to minimise the effect of diurnal variation on vascular studies.

### Macrovascular assessment

### Carotid artery ultrasound

The common carotid arteries were assessed non-invasively using a Doppler ultrasound machine with a high-resolution linear array transducer (SSD-5500 SV, Aloka, Tokyo, Japan) previously described elsewhere [25]. Briefly, subjects lay supine on an examination bed with the head turned ~45° away from the examined side. The B-mode common carotid artery images taken at the R wave of the ECG with satisfactory quality were stored in the ultrasound machine, and were downloaded later for off-line analysis. Lumen diameter and intima-media thickness (IMT) were analysed using semi-automated edge-detection software (Artery Measurement System: ver 2·02, Gothenburg, Sweden) [26]. The software automatically identified a 10 mm-long segment from the beginning of the bulb of the near wall-lumen boundary and the far wall IMT of the common carotid artery free from plaques. A plaque was defined as >50% increase in IMT compared to adjacent IMT. This automated analysis could be adjusted manually if the identification of borders was not appropriate. IMT was defined as the height between the lumen-intima and the media-adventitia borders at the far wall. Data from the right and left sides of the common carotid arteries were averaged, and mean values were used for statistical analysis for both variables.

### Brachial artery flow-mediated dilation

Brachial artery endothelium-dependent dilation (FMD) was assessed noninvasively following established guidelines [27, 28] and previously described by us elsewhere [29–31]. Briefly, subjects lay supine on an examining bed with the right arm supported and fixed in position using a positioning pillow on a metal table. The same Doppler ultrasound machine was used to obtain a B-mode ultrasound image of the brachial artery. To elicit reactive hyperaemia, an appropriately-sized cuff selected for the subject was placed around the forearm and inflated to 250 mmHg for 5 min using a rapid cuff inflation system (AI6; Hokanson, Bellevue, WA, USA). Brachial artery images were recorded for 60 cardiac cycles at baseline. The recording was restarted 30 sec before cuff deflation and continued for 3 min post-deflation. All brachial artery images were recorded and analysed by the same investigator using dedicated software (Vascular Research Tools, version 5.8.6, Medical Imaging Applications LLC, Coralville, IA, USA). FMD was calculated as the maximum percentage changes in diameter after cuff deflation compared to baseline diameter, and the time to achieve peak dilation was recorded from the time of cuff deflation.

### Transcranial doppler (TCD)

A trained researcher used an approved power-mode TCD unit with a 2-MHz probe at 100% power and a 10-mm sample volume for the examination (Nicolet Companion III; VIASYS Healthcare). A standard insonation protocol was used. An insonation depth of 45–65 mm was used to identify the M1 segment of the middle cerebral artery (MCA) through the trans-temporal window. An optimal signal was defined as one that had a clear spectral wave form and trace envelope that follow the wave form throughout the recording with no noise artefacts. Cerebral blood flow parameters including peak systolic velocity (PSV), end diastolic velocity (EDV) and mean systolic velocity (MFV) were measured according to standard protocol [32]. Static cerebrovascular resistance was estimated using the pulsatility index (PI) (PI = PSV – EDV / MFV) and the resistive index (RI) (RI= PSV – EDV / PSV). Dynamic cerebrovascular resistance was calculated by adjusting cerebral mean blood flow velocity (MFV) to central mean arterial pressure (cMAP) and is presented as adjusted cerebrovascular resistance index (ACRI) of cMAP/MFV [33].

### Pulse wave velocity (PWV) and pulse wave analysis (PWA)

Carotid-femoral pulse wave velocity was assessed non-invasively using applanation tonometry (SphygmoCor ver 8·2, AtCor Medical, Sydney, Australia), sequentially recording ECG-gated waveforms at the carotid and femoral sites. Radial artery waveforms were recorded using a high-fidelity pressure transducer, and then used to generate corresponding aortic pressure waveforms by using a generalized transfer function which has been validated previously [34]. All measurements including aortic-femoral PWV, aortic systolic BP, aortic diastolic BP, aortic mean arterial pressure (MAP) and aortic pulse pressure were made in triplicate by trained investigators and average values were used for analysis.

### Microvascular assessment

### Post-occlusive reactive hyperaemia (PORH)

Microvascular assessments were conducted as previously reported [35–37] on the dorsum of the foot. Reactive hyperaemia was assessed by ischemia-reperfusion, induced by 4 minutes of supra-systolic cuff-inflation around the ankle until pulsatile flow is terminated. Perfusion measurements were obtained for five minutes at baseline and five minutes after cuff release.

### Iontophoresis of acetylcholine (Ach) and sodium nitroprusside (SNP)

This method has been described elsewhere [38]. Laser Doppler Perfusion Imaging (LDPI, MoorLDI, Moor Instruments, Axminster, UK) was used to measure the cutaneous flux response to the iontophoretic delivery of Acetylcholine (ACh, endothelium dependent response) and Sodium Nitroprusside (SNP, endothelium independent response). ACh (1% ACh in mannitol, Michol-E, Bausch and Lomb Inc, Germany) or its control (3% mannitol in water) was delivered using an anodal current (5 x 0·1 mA for 20 seconds with a 60 second interval between each dose, resulting in a total charge of 10mC) (DRT4, Moor Instruments, Axminster, UK). SNP (0·25%: 25 mg/ml Nitropress, Hospira, USA in 0·45% NaCl w/v saline, Baxter UK, Norfolk, UK) or control vehicle (0·45% NaCl) was delivered using a cathodal charge (1 x 0·2 mA for 60 seconds, resulting in a total charge of 12 mC). Cutaneous flux was assessed by LDPI at rest and then every 20 seconds following the start of the iontophoresis charge for a total of 7 mins with Ach and 6 mins for SNP. The mean cutaneous flux was determined over an area of 0·78 cm^2^ (Moor software). The data are expressed as peak and total response (area under the curve, normalised for resting blood flux).

### Magnetic resonance imaging (MRI)

An MRI scan of the brain was performed on a different visit in order to calculate WMH volumes. All MRI images were acquired using a 1·5 Tesla scanner (Philips, The Netherlands) using an 8 channel head coil. After initial survey images T1 structural images of the brain with an axial oblique orientation were acquired. All images were examined for quality.

Cortical reconstruction and volumetric segmentation was performed with the Freesurfer image analysis suite (Freesurfer version 5·1·0, Athinoula A. Martinos Center for Biomedical Imaging, Charlestown, Massachusetts), which is documented and freely available for download online (http://surfer.nmr.mgh.harvard.edu/). All Freesurfer images were examined by an independent researcher for accurate volume reconstruction and correction of errors.

### Statistical analysis

Data were treated as continuous variables wherever possible to maximize power. All normally distributed data are presented as mean ± SD. Skewed data were appropriately log transformed and presented as geometric means (95% CI). Statistical significance for categorical variables was calculated using the chi-squared test and the Student’s t-test for continuous variables. WMH volume was treated as a continuous variable in all study participants. A linear regression model was used to explore the association between cerebral WMH and variables of interest. To explore independent determinants of associations between variables, multinomial logistic regression analysis was used. Age, sex and blood pressure (MAP) were forced into the final model, as these all have an established effect on cerebral vascular autoregulation. To assess the best vascular measurement (microvascular, macrovascular, endothelial and cerebral blood flow parameters) that predicts WMH volume we used a multi variable regression model. Alpha was P≤0·05. In keeping with the recommendations of Cupples (Cupples et al., 1984) and Rothman (Rothman, 1990) for performing hypothesis driven analyses, significance of the variables of interest is reported without adjustment for multiple testing. Any additional variables in these models (although not reported here) should be subject to such Bonferroni correction. Statistical analysis was performed using Stata SE 13·0 (Microsoft version: StataCorp Ltd. TX, USA).
